# Contact-induced polarization shapes HIV-1 infected T cells for efficient HIV-1 dissemination at the virological synapse

**DOI:** 10.1186/1742-4690-10-S1-P38

**Published:** 2013-09-19

**Authors:** Elisabetta Groppelli, Shimona Starling, Clare Jolly

**Affiliations:** 1 University College London, London, UK

## Background

Transmission of HIV-1 between CD4+ T cells is by cell-free infection or direct cell-to-cell spread at intercellular junctions called virological synapses (VS). Transmission at VS has been shown to be orders of magnitude more efficient than cell-free infection. Increasing evidence suggests a key role for cell-cell spread in HIV-1 dissemination, replication and pathogenesis. Our previous work showed that HIV-1 VS formation is associated with striking organelle polarization to intercellular junctions and that a large volume of the synaptic cytoplasm in the infected cell is occupied by mitochondria. However, whether this occurs in response to contact and the functional consequences imparted by this are unclear. Therefore, we set out to investigate the potential role of mitochondria in the formation of the HIV-1 VS and in HIV-1 cell-cell spread.

## Materials and methods

A combination of methods that include fixed and live cell imaging, siRNA and pharmacological inhibitors was used in this study.

## Results

Using immunofluorescence staining we found a strong association between Gag enrichment at the VS and co-polarization of mitochondria within the infected cell. Live cell imaging showed that mitochondria polarization in the infected cell occurred within minutes of establishment of contact with the target cell and was concomitant with that of Gag. Perturbation of mitochondria trafficking and function with siRNA and pharmacological inhibitors impaired not only Gag-mitochondria co-polarization, but also HIV-1 cell-cell spread, demonstrating a functional role for mitochondria recruitment to T cell junctions during HIV-1 dissemination. In particular, inhibition of mitochondrial ATP synthesis and Ca^2+^ buffering had a strong effect on HIV-1 cell-cell spread. This effect was significantly stronger in cell-cell spread than in cell-free spread, supporting a specific role for mitochondria in HIV-1 spread at the VS. Additionally, all the known component required at the plasma membrane for the formation of the HIV-1 VS (Env and CD4 and LFA-1 and ICAM-) were also required for Gagmitochondria co-polarization to the VS, suggesting that stable cell-cell contacts and localized outside-in signaling direct T cell remodeling and HIV-1 spread.

## Conclusions

Taken together, our data support a model in which formation of stable interactions between the infected and uninfected cell at the VS induces cytoplasmic remodeling within the HIV infected cell, leading to T cell polarization and the translocation of mitochondria to the contact zone. In this model, HIV-1 infection, coupled with physical contact between infected and uninfected cell, directs reorganization of cytoplasmic organelles to favor its own dissemination.

